# Emergent technological variation in archaeological landscapes: a primate perspective

**DOI:** 10.1098/rsif.2023.0118

**Published:** 2023-06-21

**Authors:** Jonathan S. Reeves, Tomos Proffitt, Suchinda Malaivijitnond, Lydia V. Luncz

**Affiliations:** ^1^ Technological Primates Research Group, Max Planck Institute for Evolutionary Anthropology, Leipzig, Germany; ^2^ Center for the Advanced Study of Human Paleobiology, Department of Anthropology, The George Washington University; ^3^ Department of Biology, Faculty of Science, Chulalongkorn University, Bangkok, Thailand; ^4^ National Primate Research Center of Thailand, Chulalongkorn University, Saraburi, Thailand

**Keywords:** primate archaeology, long-tailed macaques, percussive technology

## Abstract

Archaeological evidence informs our understanding of the evolution of hominin behaviour. Such evidence is traditionally used to reconstruct hominin activities and intentions. In the Plio-Pleistocene, the presence or absence of specific tools and variation in artefact density is often used to infer foraging strategies, cognitive traits and functional activities. However, the Plio-Pleistocene archaeological record is known to be time-averaged and forms through the aggregation of repeated behavioural events over time. Thus, archaeological patterns do not reflect discrete episodes of activity, but rather the interaction of behaviour with environmental factors over time. However, little is known about how such interactions produce archaeological variation diversity. Primate archaeology can help address this research gap by providing the opportunity to observe how behaviour produces material patterns in a natural setting. This study, thus, examines how varying the material properties of stone and resource availability influence the artefactual signature of nut-cracking in a population of long-tailed macaques from Lobi Bay, Yao Noi island, Thailand. Results show that these interactions can produce a structured and diverse material signature in terms of artefact density and frequency of specific artefact types. These findings demonstrate how material patterns can emerge from long-term interactions between behaviour and environmental factors.

## 1. Introduction

Stone artefacts are one of the primary means through which hominin behavioural evolution is investigated [1–8]. This is done by connecting the variation that is observed in aspects of the archaeological record to specific behavioural processes [9,10]. As a result, the ways in which frequency and representation of specific types and technological attributes of stone tools vary form the basis for behavioural inference in the Early Pleistocene [11]. When combined with landscape-scale palaeo-environmental data, patterns in lithic assemblages are argued to reflect the ecological underpinnings of stone tool use and ultimately the adaptive strategies of our hominin ancestors [12].

Within the Early Stone Age (ESA), there is an ongoing discussion regarding the range of behavioural and environmental processes that probably contribute to the variation observed in the archaeological record [1,13–17]. For example, variations in the density of stone artefacts across space are argued to reflect how hominins organized themselves in space [17–21]. Foley [22] discussed how the spatial organization of various tool behaviours would influence the density of material across the landscape. As such, the identification of localized concentrations of artefacts has often been interpreted as central places that served as localities where hominins aggregated food resources [23–25]. However, others have suggested that this pattern arises simply due to using tools in particular environmental contexts [15]. The differences in the representation of tool types between sites have also been used as evidence that hominins carried out different behaviours in different places [26,27]. Potts [28] discussed how different activities, such as butchery, influenced the representation of different tool types such as large cutting tools at a given site. However, others have suggested that these patterns could arise due to differences in skill or even the species of hominins making the tools [11,29]

There is also debate about the degree of tool variation that reflects the technical know-how of hominin tool makers and the social transmission of information versus the constraints imposed by the broader environmental constraints [16]. For example, the variation in the representation of specific core forms is often associated with specific tool production strategies possibly related to the social transmission of information [14,16,30]. Whereas others have emphasized the mechanical properties and availability of raw materials in shaping inter-site variation in stone tools [31–34]. Moreover, it has been suggested that the interaction of behaviour such as tool manufacture and transport with broader environmental processes gives rise to the variation observed in the Oldowan archaeological record [15,31,35,36]. It is now known that four divergent species of non-human primates use and transport stone tools to gain access to a variety of terrestrial and marine food resources, producing a durable material record that can be studied from an archaeological perspective [37–39]. Therefore, primate material culture has become increasingly used as a referential model for understanding ancient hominin behaviour.

However, some have questioned their applicability as models for hominin evolution [40] citing their distinct evolutionary history, differing anatomy, foraging behaviour and lived-in environments [40,41]. Others have highlighted differences between primate percussive tools and hominin core and flake technology to argue that the primate analogy is limited in its explanatory power [42,43].

Nevertheless, the value of primate archaeology lies not within the few similarities between modern tool-using primates and hominins, but in its ability to connect behavioural processes with a tangible material end product [42]. Combining behavioural observation with the systematic documentation of emerging primate stone assemblages allows us to establish robust connections between stone tool behaviour and the material variation it produces. In addition, because primate stone tool use can be observed in the wild, primate archaeology also affords the opportunity to investigate how the intersection of tool-using behaviours, broader social contexts and ecological factors such as resource availability creates diversity in their material record [39,44–48]. For example, percussive tools used by long-tailed macaques (*Macaca fascicularis*) in Thailand have been shown to vary in size, shape and damage patterns according to the foods being processed [48]. Inter-group variation in hammerstone damage patterns has also been linked to social processes governing how specific tools are re-used [47].

Environmental assessments of chimpanzee nut-cracking sites from West Africa have also shown how raw material properties influence both the development of damage and the accumulation of artefacts [44,49]. Furthermore, when percussive behaviours are undertaken on naturally occurring fine-grained isotropic materials among both macaques and capuchins there is a high chance of the formation of accidental sharp-edged flake assemblages [45,50]. Such studies are relevant to broader discussions in hominin behavioural evolution as they highlight the material diversity associated with various percussive behaviours. There is a growing interest in Plio-Pleistocene percussive technology as it may have been the precursor to core and flake technology [51–56]. Thus, these studies provide a means by which to better detect the signatures associated with such behaviours in the Plio-Pleistocene and refine the behavioural inferences we draw from them.

Beyond their implications for the hominin archaeological record, the insights gained through the study of wild primate material culture can be generalized to discuss broader relationships between material patterning and behavioural inference. Studies of chimpanzee tool use have shown that the transport and discard of stone at nut-cracking sites produces localized concentrations of lithic material argued to be like those found in the Oldowan [54,57,58]. Moreover, landscape surveys of capuchin material culture at Fazenda Boa Vista, Brazil have shown how nut-cracking leads to a heterogeneous landscape of stone hammers and anvils [59]. In the Taï Forest (Cote d'Ivoire), the cumulative effects of individual chimpanzee nut-cracking and short-distance transport have been shown to produce non-random spatial patterns where tools decrease in mass as the distance from where they naturally occur increases [46]; a pattern that has often been documented in Oldowan contexts [21,60,61]. Therefore, primate studies provide insights into the range of potential behaviours that can produce material patterning that we encounter in the archaeological record.

Still, some have criticized the use of primate stone tool use and more broadly middle range approaches because they do not match the quality of the archaeological record [62]. With regard to the Plio-Pleistocene, the majority of the archaeological record represents a time-averaged signature of repeated tool discard events as opposed to the discrete behavioural episodes often observed in ethnographic and ethological studies [63,64]. Therefore, there is a temporal mismatch between our interpretive frames of reference and the time-depth present in the archaeological record [63]. While computational simulations have begun to reveal the long-term implications of behaviour and the environment on record formation [35], there is little opportunity to investigate the influence of such formation processes in a natural setting.

Stone tool using long-tailed macaques of Southern Thailand [37], however, provide an opportunity to study the relationship between repeated stone tool use, environmental factors, and the formation of a material record, as they use tools in a variety of distinct environments [37]. In particular, the long-tailed macaques of Yao Noi Island, crack nuts in an abandoned oil palm (*Elaeis guineensis*) plantation that provides a resource-rich landscape varying in the availability and quality of the available stone [65,66]. The spatial configuration of the available resources and stone materials allows the macaques to re-use the same locations for nut-cracking, resulting in a highly visible, landscape-wide material record [50]. In addition, the variation in raw material properties across the study area produces a diverse archaeological record ranging from an abundance of fragments to assemblages containing a large proportion of accidentally produced flakes and flaked pieces [50]. Environment assessments show that the distribution of palm trees varies, providing an opportunity to investigate how landscape-scale variation in raw material quality and resource availability influences the formation of a material record.

Here, we report on the landscape-wide material signature of nut-cracking behaviour associated with long-tailed macaques of Yao Noi Island. We used landscape archaeological methods to conduct a systematic survey of the nut-cracking assemblages, nut trees and the available raw materials across the study area. These data allow us to characterize the diversity of nut-cracking assemblages and investigate the relationship between behaviour, environmental factors and the formation of stone assemblages. We were able to identify the aspects of macaque behaviour and tool use that are crucial to the emergence of a landscape-scale material record and the aspects of the environment that influence it. Thus, the results of this work show how the interaction of a single behaviour (nut cracking) with varying resource density and stone availability, over time, creates lithic assemblage diversity at the landscape scale. The patterns documented by this work are then generalized to discuss the role of long-term interactions between behaviour and the environment in the emergence of archaeological patterns in the Plio-Pleistocene.

## 2. Material and methods

### 2.1. Materials

#### 2.1.1. Study area

**Figure 1.**
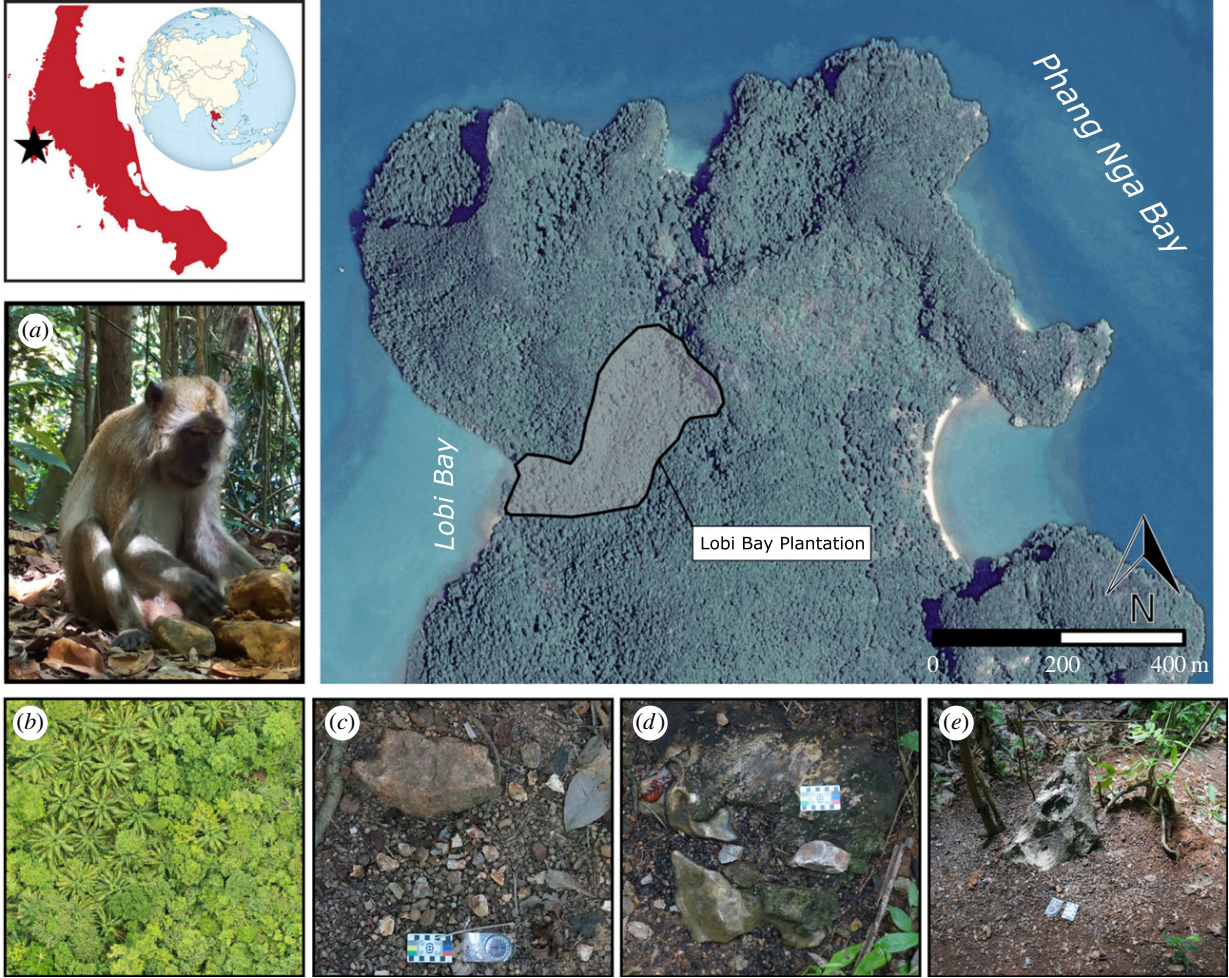
Map showing the geographical context of Ao Lobi Bay plantation (ALP). This map was produced using the package ggmap in R v. 3.04 [67]. Insets: (*a*) An example of a long-tailed macaque cracking nuts with a stone hammer and anvil. (*b*) An example of how identifiable palm trees are from aerial imagery of the forest canopy. (*c*) Example of a nut-cracking site comprising primarily low-quality (red-oxidized limestone) material. The soft nature and presence of internal fracture planes produce angular debris. (*d*) Example of a nut-cracking site comprising high-quality (grey limestone) material. (*e*) Example a semi-exposed limestone boulder used as an anvil.

Lobi Bay is situated in the northern part of Yao Noi Island, in Ao Phang Nga national park, Thailand (figure 1*a*). The site consists of an intertidal zone with a rocky beach, where long-tailed macaques have been observed using stone tools to exploit sessile and motile marine wildlife including crabs, oysters and sea snails [47,68]. Beyond the shoreline exists a dense coastal forest containing a now-abandoned oil palm nut plantation of 0.051 km^2^ in size (henceforth Ao Lobi plantation site or ALP). Since the plantation ceased operation and was abandoned in the early 2000s, a group of long-tailed macaques has begun exploiting the remaining oil palm trees by cracking the fruited nuts. This is done by transporting stones to be used as hammers, over short distances, to semi-exposed boulders or large cobbles that serve as anvils [65,66]. The oil palm nut is then placed on the anvil and struck with the hammer to break through the outer shell and access the edible kernel of the nut. During this process, mishits resulting in collisions between the stone hammer and anvil lead to fracture and result in the accumulation of debris in the form of fragments, flakes and large quantities of cracked palm nut shells [50]. Once the macaques have finished nut cracking, hammerstones are often discarded at the anvil, further contributing to the accumulation of stone at the site.

**Figure 2.**
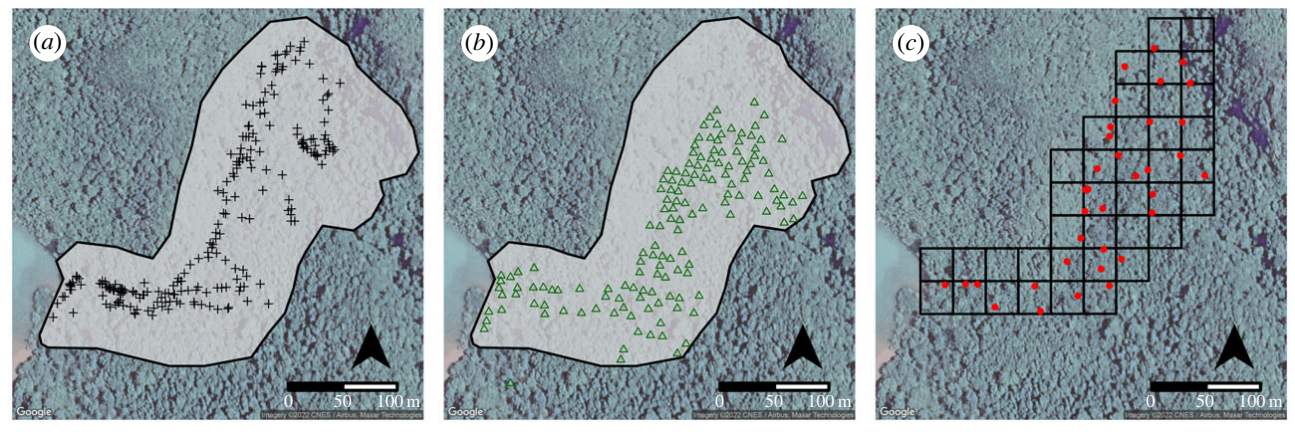
Maps showing locations of sampled materials. (*a*) Locations of documented nut-cracking sites across the study area. (*b*) Location of palm trees across the study area. (*c*) Map of stratified random sampling strategy and locations of sampled nut-cracking sites. All maps were produced using ggmap in R v3.04 [67].

The geography of the old plantation is particularly amenable to nut cracking as the underlying geology also provides an abundance of stone material suitable for the behaviour. ALP is situated in the centre of an asymmetrically shaped ravine that is framed by sources of stone on each side (figure 1*b*). The north-northwest side of the ravine is a strip of flat forest floor which is abutted by cliffs of limestone to the northwest. The process of weathering and erosion causes the cliffs to fracture, providing an abundance of potential hammers and anvils, in the form of irregularly shaped cobbles, and semi-exposed boulders at its base. The south-southeastern side of the ravine consists of a gentle slope. Though stone is less abundant, erosional processes expose an oxidized red limestone from the slope that is also ranging from small cobbles to small boulders. A large number of palm trees in combination with the abundance of suitable stones permits nut cracking across the entirety of ALP (figure 2*b*).

The stationary nature of most anvils causes the local macaque population to return to and re-use the same locations over time. During the 2021 and 2023 field seasons, the long-tailed macaques were observed using the anvils for nut cracking over multiple days. In general, the macaques would arrive at ALP around mid-morning to crack nuts until the early afternoon before leaving. This pattern of behaviour was observed to repeat over multiple days. These observations were augmented by camera trap footage documenting the macaques returning to the same anvils at multiple independent instances to crack nuts (electronic supplementary material, SOM 2, SOM 3). In 2023, a survey of previously documented sites (April 2021) found that new artefacts had accumulated at locations where the authors had previously collected the material. While the total duration of time represented in these assemblages is unknown, the accumulation of material appears to occur over the span of multiple years.

**Figure 3.**
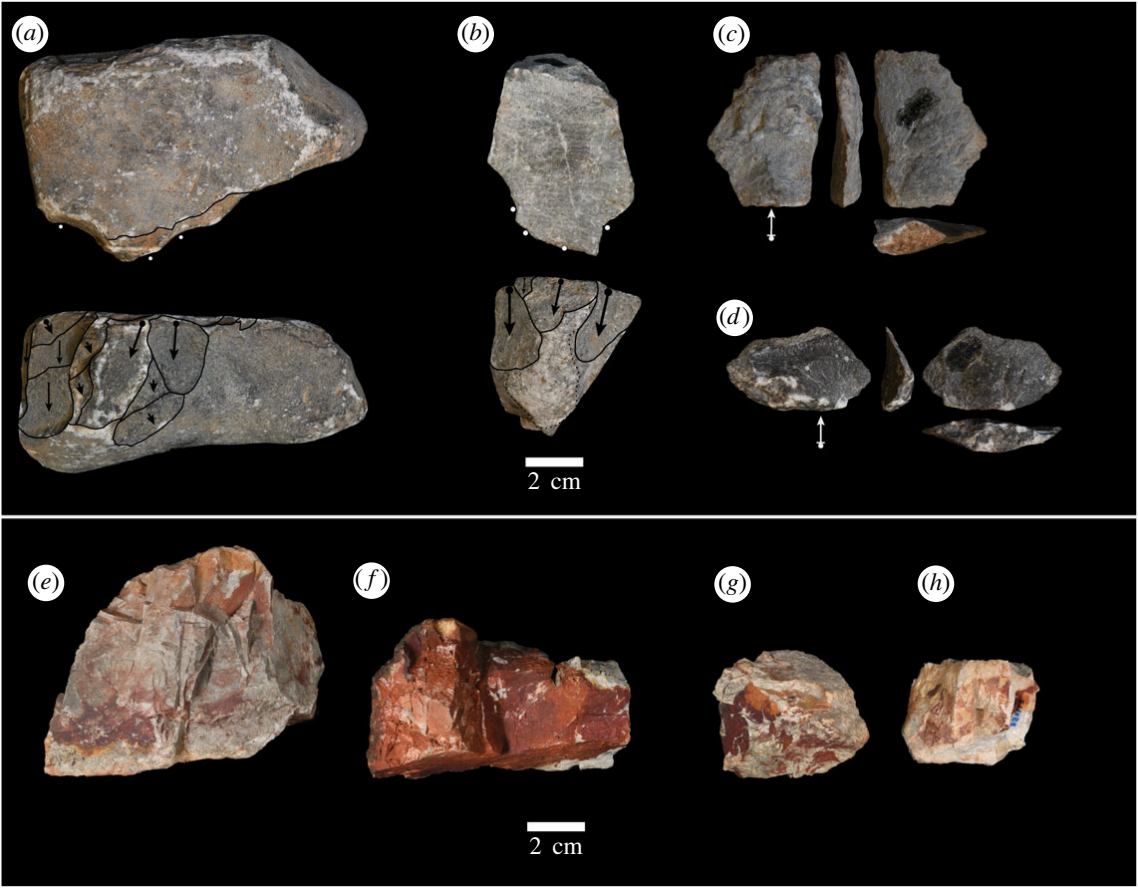
Examples of lithic materials associated with each raw material type. (*a*–*d*) Light grey limestone; (*e*–*h*) red-oxidized limestone. (*a*) A light grey limestone hammerstone showing signs of battering and negatives associated with conchoidal fracture. (*b*) A hammerstone fragment bearing traces of battering and previous scar removals. (*c*,*d*) Examples of flakes detached during nut-cracking events. (*e*) Red-oxidized limestone hammerstone. (*f*) Example of an angular fragment associated with nut cracking. (*g*) An angular fragment bearing traces of battering due to nut cracking.

The varying material properties of available stone material and the density of palm trees vary across the study area, thus allowing us to examine how the interacting effects of site reuse, raw material quality and resource distribution produce material patterning. The stone derived from the limestone cliffs on the north-northwest side of the ravine is silicified and isotropic (figure 3). As a result, the material is resistant to breakage, permitting continuous reuse of the stone. Though breaks are probably infrequent, the isotropic nature of the rock causes breaks to produce fragments that range from angular shatter to those bearing evidence of conchoidal fracture [50]. Some of which are identical to products that are produced during intentional knapping [50]. In some instances, natural inclusions of a grey finer-grained limestone can be found within the high-quality grey limestone.

By contrast, the limestone on the south-southeast side of the valley possesses an oxidized reddish exterior that is a buff or cream colour on the inside (figure 3). Examination of the physical properties of the material in the field indicates that it is considerably softer and possesses a multitude of perpendicularly running internal fracture planes. As a result, the soft and flawed nature of the red-oxidized limestone causes it to frequently break during use, resulting in the accumulation of angular fragments. The distinct variation in the quality of limestone across the study area allows us to examine how the reuse of sites and mechanical properties of stone affect the material signature of nut cracking at a landscape scale.

Field observations also suggest that geomorphological processes such as slope and precipitation have minimal influence on the formation of nut-cracking assemblages at ALP. Although ALP is situated within a ravine, most assemblages are situated on flat-lying surfaces, indicating that gravity-driven processes such as downslope displacement have had minimal influence on the representation of artefacts within the sampled assemblages. Some of the assemblages sampled from the S-SE side of the ravine are, however, situated on gentle slopes, resulting in some lithics found slightly downslope from the anvil. However, most of the lithic debris and broken nutshells remain within close association with the anvil, suggesting that such downslope displacement is minimal over time.

ALP and Thailand more broadly are situated within a tropical region and have a well-defined rainy season. As a result, the assemblages are subject to heavy rains on an annual basis. Nevertheless, hydrological influences such as run-off from precipitation appear to have minimal effect. This is due to the densely vegetated nature of the forest limiting the influence of widespread sediment transport and erosion. Moreover, most assemblages possess a fraction of stone and nut debris that is 1 cm or less in size suggesting that the winnowing effects of flowing water are minimal or non-existent (figure 1).

### 2.2. Methods

To investigate how factors such as site reuse and material properties influence the formation of nut-cracking assemblages, we used landscape archaeological approaches to document and describe the variation in the material properties of available stone, the number of palm nut trees, and the composition of nut-cracking stone assemblages across the study area.

#### 2.2.1. Documenting variation in stone quality across space

Anvils were used as a proxy for how the mechanical properties of available materials naturally vary across space. Since smaller stones are often used as hammers, they are more susceptible to transport due to tool use and, thus, may provide a distorted view of the natural availability of stone. Since most anvils are large cobbles or semi-buried boulders, they represent fixed points on the landscape and therefore mimic the underlying geological variation within the study area, and therefore more accurately reflect the natural availability of stone within the study area. The locations of 221 anvils were recorded in real-world coordinates using an Emlid Reach RS-2 real-time kinematic system (RTK) (figure 2*a*).

Each anvil was classified as one of two raw material types according to its physical appearance: fine-grained grey limestone and red-oxidized limestone. Fine-grained grey limestone refers to isotropic, silicified limestone that is derived from the limestone cliffs in the north-northwest of the study area. Red-oxidized limestone refers to stone predominately found on the south-southeastern side of the study area.

In addition, the material properties of each stone anvil were also quantified using a Proceq digital Schmidt hammer to measure rebound hardness following Braun *et al*. [34]. Rebound hardness is an ideal measure for quantifying the quality of percussive tools as it is correlated to a rock's ability to withstand strain [69]. Therefore, it is likely to be correlated with how likely a rock is to break when used for percussive activities. Rebound hardness is also sensitive to the presence of flaws or impurities within the rock [34]. Therefore, rebound hardness should be sensitive to the observed material properties of the two rock types at ALP. To estimate the hardness for each anvil, 10 rebound hardness measurements were collected and then averaged to ensure that the measurement was accurate.

#### 2.2.2. Documenting the locations of palm trees

The locations of oil palm trees were mapped from an orthomosaic image of the forest canopy generated using an unmanned aerial vehicle (UAV). Palm trees show a very distinct canopy compared with local trees which allowed us to map the location of every palm tree across the study area (figure 2*b*). The ortho-mosaic was generated using images captured by a DJI Mavic 2 quadcopter 30 m above the forest canopy at a speed of approximately 5 m s^−1^. The UAV was flown along 18 line transects spaced approximately 8–12 m apart. The camera was oriented orthogonally to the forest canopy [70] and set to capture an image every 2 s. This resulted in 779 images with an image overlap of 85%–95%. The images were processed and stitched together using Agisoft Metashape Professional (v. 1.6.5), producing a georectified orthomosaic with a resolution of 1.4 cm/pixel. The location of each palm tree was then manually identified and recorded using the centre of the crown of each tree as an ESRI point file in QGIS (v. 3.20).

#### 2.2.3. Documenting nut-cracking assemblages

We sampled stone assemblages from 34 nut-cracking sites across the study area. Nut-cracking sites were defined as locations that possessed battered elements (i.e. stone with evidence of being used as hammerstones), an anvil and broken nutshells. Nut-cracking assemblages were sampled following a stratified random sampling strategy to ensure that bias did not influence our data collection protocol (figure 2*c*). The study area was divided into a grid consisting of 30 × 30 m^2^. Each 30 × 30 m^2^ was further subdivided into 5 × 5 m^2^. A random number generator was used to select a 5 × 5 m^2^ within each 30 × 30 m^2^ to be comprehensively surveyed for nut-cracking sites. If no nut-cracking site was present within a 5 × 5 m^2^, another location was chosen at random. In cases where more than one anvil was present, the first anvil encountered or identified was selected for sampling.

The location of each site was then recorded in real-world coordinates using the RTK system. A 1 × 1 m^2^ was then drawn with the anvil in the centre. A sampling unit of this size was chosen as the majority of nut-cracking material is concentrated within a 1 m^2^ of the anvil [65]. In some cases, the square was offset from the centre of the anvil to mitigate the minor effects of slope and anvil size on the distribution of nut-cracking material. For example, in cases where the anvil was the edge of a larger boulder spanning multiple metres in size, the centre of the square was adjusted so that the damaged portion of the anvil and most of the lithic scatter was included within the 1 × 1 m^2^. Stone artefacts were then collected from within the 1 × 1 m^2^ surrounding the anvil.

#### 2.2.4. Estimating raw material quality and availability across Ao Lobi plantation

The hardness values from mapped anvils were used to estimate the spatial distribution of rebound hardness across the study area using Kriging interpolation. This provided a means by which to examine whether sampled nut-cracking assemblages were influenced by the quality of the local raw materials. Kriging is a commonly used technique, used extensively in geography, ecology, exploration geology and archaeology, that is used to estimate the value of a variable at a given location where the value is not known [71–73]. Unlike other interpolation methods, kriging considers the spatial structure of a dataset to determine its estimates [71,72]. As a result, it often provides more accurate predictions than methods such as inverse distance weighting [72]. Here, we use ordinary kriging to estimate the hardness of locally available material at a resolution of 1 m. Kriging was carried out using the gstat package [74] in R (v. 4.1.2) [75].

#### 2.2.5. Estimation of palm tree availability

To estimate the availability of trees across the study area and for each assemblage, we calculated the density of palm trees across space using a kernel density function. Kernel density was calculated using R (v. 4.0.3) using the spatstat package [76]. In addition, to understand the effect of palm tree locations on the formation of stone assemblages, we calculated the mean distance from a palm tree for each assemblage. Distance to palm trees is probably an important factor in stone assemblage formation in ALP given that they provide the food resource for which nut-cracking is used. Sites with shorter average distances would be situated closer to a greater number of palm trees and therefore may be used more often than other sites.

#### 2.2.6. Characterization of stone assemblages

Lithic analysis protocols follow Proffitt *et al*. [50]. The technological diversity of the lithic assemblages recovered from ALP was published by the authors in [50]. As such, the analysis presented here focuses on describing landscape variation in the material signature of nut cracking at the assemblage scale. The following is a summary of the analysis undertaken. A full description of the methodology is provided as electronic supplementary material (SOM 1).

Artefacts sampled from each nut-cracking assemblage were characterized according to a series of categorical and quantitative attributes. Each artefact was classified as a hammerstone, anvil, flake or angular fragment. Each artefact was assigned one of two raw material types according to its physical properties: fine-grained grey limestone, and red-oxidized limestone (figure 3). In addition, length, width, thickness and mass were recorded for each artefact. These data were then summarized at the assemblage level to quantitatively characterize the material composition of each assemblage.

Each assemblage was characterized in terms of its density, artefact composition and raw material diversity. Artefact density was calculated as the number of artefacts recorded within the 1 × 1 m^2^. Assemblage composition was characterized according to the proportion of hammerstones, the proportion of angular fragments, the proportion of flakes, the proportion of pieces bearing evidence of conchoidal fracture (flakes and hammerstones bearing flake scars) as well as the proportion of pieces possessing evidence of battering present within the assemblage. In addition, the diversity of raw material present within an assemblage was calculated as the number and proportion of fine-grained grey limestone and red-oxidized limestone material within each assemblage.

#### 2.2.7. Statistical analyses

To examine the influences of raw material quality and palm-tree distribution on assemblage variation, a series of statistical comparisons were conducted. First, differences in raw material quality were assessed using a Mann–Whitney *U-test* to compare rebound hardness values of anvils of different materials. Mann–Whitney *U* was chosen over the parametric *T*-test because hardness values are not normally distributed (Shapiro–Wilk: *p*-value = 4.52 e-07). The level of statistical significance was determined using an α value of 0.05.

We used a generalized linear model (GLM) with a negative binomial error structure to investigate the effect of raw material availability and nut-tree availability on the accumulation of nut-cracking artefacts within an assemblage. The response variable was defined as the number of artefacts within a 1 × 1 m square. Although a GLM with a Poisson error structure is preferred when dealing with count data, we choose to use a negative binomial model to mitigate issues of overdispersion in the response variable [77]. The average distance from palm trees and rebound hardness—as estimated by the kriging interpolation—were included as fixed effects. To establish the significance of the full model we used a likelihood ratio test to compare its deviation with a null model comprising only the intercept. The significance of each variable included in the model assessed by comparing the full model to reduced models using a likelihood ratio test.

To examine the overall effect that raw material availability had on the representation of specific rock types at sites, we constructed a second model, with the proportion of high-quality raw material as the response variable and average distance to palm trees and hardness as the predictors. A binomial error structure with a logit link function was chosen because the response variable is a ratio thus constraining values between 0 and 1 [60]. A likelihood ratio test was used to establish the significance of the full model by comparing it with a null model. As with the negative binomial model, the significance of each variable included in the model assessed by comparing the full model with reduced models using a likelihood ratio test. During analysis, we checked various diagnostics of model validity and stability using variance inflation factors, leverage and DFBetas. None of these tests indicated obvious influential cases or instability.

Finally, to investigate the effect of raw material properties and palm nut availability on the formation of nut-cracking assemblages, we compared the composition of each sampled assemblage with hardness and palm nut availability using a correlation test. Kendall's tau [78] was used to compare the number of battered pieces, hammers, fragments, flakes and flaked pieces compared with hardness and average distance to palm trees separately. Kendall's tau was chosen over a parametric correlation coefficient because these data are non-normally distributed. All analyses were carried out using R (v. 4.3.0) [75].

## 3. Results

### 3.1. Environmental data

#### 3.1.1. Hardness of available stone

**Figure 4.**
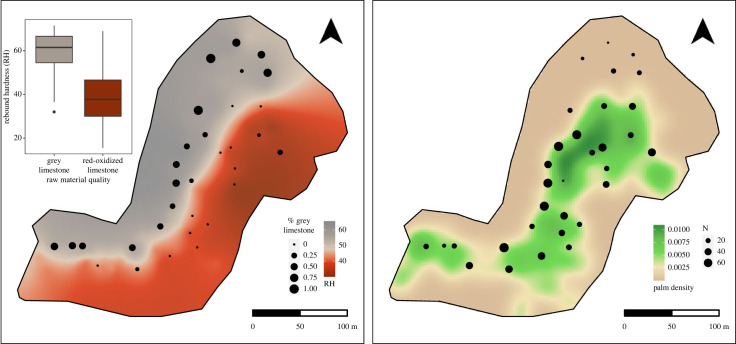
Spatial distribution of raw materials and palm trees. Left: kriging interpolated map comparing the distribution of high- and low-quality limestone across the study area with the proportion of high-quality material within each sampled nut-cracking assemblage. Each point represents a sampled nut-cracking assemblage. Right: kernel density map comparing the density of palm trees across the study area with the total number of artefacts documented at each sample location.

An analysis of the hardness of available stone indicates that the two raw material types have distinct hardness signatures. The fine-grained grey limestone is significantly harder than the red-oxidized limestone (Mann-Whitney *U*: *W*= 11 894, *p*-value < 0.0001, figure 3*b*). The fine-grained grey limestone has a mean rebound hardness value of 60.0 whereas the red-oxidized stone has a median hardness of 39.08 (figure 4, left). These differences are probably driven by not only variation in the hardness of the two rock types but also the presence of internal flaws present in the red-oxidized limestone. Moreover, the interquartile ranges associated with each material type do not overlap. These results attest to the observed differences in the fragility of the raw material and show that specific hardness values can be reliably associated with the rock types. Thus, variation in hardness values across the study area can be used as a proxy of available stone quality.

The results of the kriging interpolation show that the availability of these two rock types is also highly structured across the landscape (figure 4, left). These results show that locally available materials on the north-northwest side of the ravine are substantially higher than those on the south-southeast side. The estimated hardness values of locally available materials in the north-northwest region rarely fall below 50, indicating that the isotropic fine-grained grey limestone is primarily available on this side of the ravine. By contrast, the south-southeast region of lower-quality material contains values of 40 and less showing that this area is dominated by the more breakable, red-oxidized material (figure 4, left). A small gully in the northern region of the south-southeast side, however, contains a small number of semi-exposed fine-grained grey limestone boulders.

**Figure 5.**
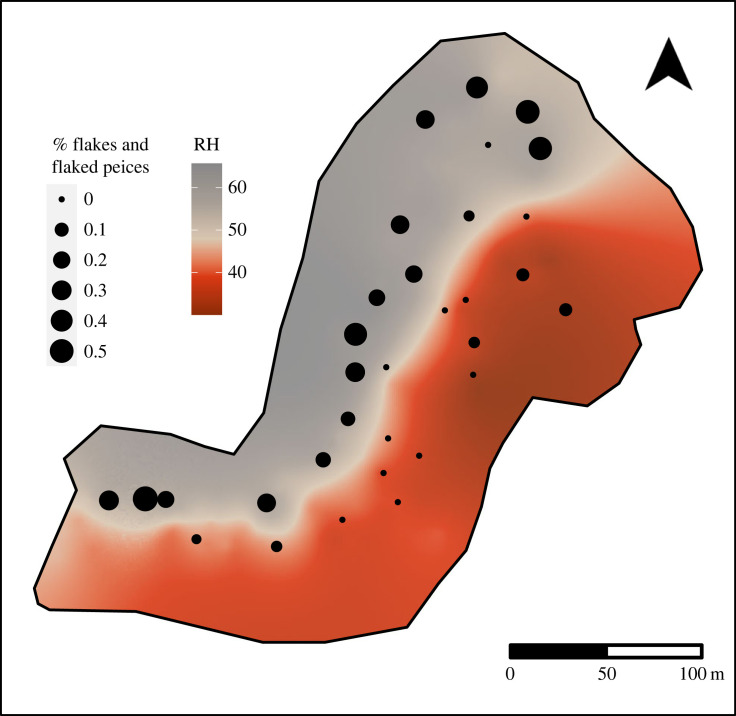
The influence of hardness on the representation of flakes and flaked pieces.

The area towards the ravine's central axis rapidly converges on intermediate values around 48 (figures 4 and 5). However, this does not indicate that there is a continuous hardness gradient between the two types of rocks. It is evident, from field observations and the analysis of the stone assemblages that no intermediary type of stone exists. Rather areas with intermediate hardness values can be interpreted as a zone of admixture where varying proportions of fine-grained grey limestone and red-oxidized limestone are found (figure 2*a*).

In terms of resource distribution, palm trees are abundant across the study area but do vary in their density. The distribution of palm trees straddles the central axis of the ravine (figures 2*c* and 4) facilitating the availability of oil palm nuts in both raw material zones. Palm trees are most concentrated in areas immediately abutting the limestone cliffs to the northwest (figure 4). Furthermore, palm trees are found in lower densities to the west near the shoreline and extend up the shallower slope of the south-southeastern side of the ravine (figure 2*b*).

### 3.2. Summary of archaeological data

Analysis of the lithic assemblage indicates that nut-cracking produces a widespread and heterogeneous material signature across the landscape (electronic supplementary material, SOM 2). While the mean number of artefacts recovered from a single square metre is 35, artefact densities range from as few as 10 to as many as 74 m^−2^. The representation of raw materials within a given assemblage ranges dramatically from cases where a single raw material is present to those that exhibit varying levels of admixture. The number of hammerstones ranges widely as well (min = 0, max = 17) comprising from 0% up to 38% of an assemblage. Nut-cracking lithic debris comprises the largest proportion of material recovered from all sites, with the proportion of fragments ranging from 62% and 100% of an assemblage. The number of flakes or flaked pieces also ranges widely, comprising anywhere between 0% and 57% of an assemblage. Moreover, the proportion of pieces bearing traces of battering ranges between 2% and 85% of the assemblage.

### 3.3. Environmental influences on assemblage formation

The results of our analysis show that much of the variation across archaeological assemblages can be correlated with environmental factors in the landscape (electronic supplementary material, SOM 3). With respect to artefact density, assemblages with the greatest number of artefacts are those located in areas where palm trees are dense (figure 4, right). Moreover, they occur in areas where the estimated hardness values of locally available stones are high. Furthermore, the results of the negative binomial GLM indicate that only the average distance to nut-bearing trees has a significant interacting effect on the number of artefacts recovered within a single square metre. The full model was highly significant when compared with the null model (*θ* = 341.69, d.f. = 29, *p*-value < 0.001). Specifically, there was a significant negative effect on the average distance to palm trees and the number of artefacts present (estimate: −0.013, *X*^2^ = 48.967, *p*-value = 4.52×10^−5^). Assemblages located in areas of ALP where palm trees are less dense are more likely to possess fewer tools. Although there was also positive effect of the hardness of locally available material and the number of artefacts in an assemblage, this effect was not significant (estimate: −0.007, *X*^2^ = 48.967, *p*-value = 0.376). This result implies that the hardness of the available stone does not influence the number of artefacts recovered from a given location. This result is counterintuitive because the hardness of the raw material—its ability to resist breaking—determines how often a percussive tool will break, producing lithic debris. These results may indicate that site reuse plays a critical role in assemblage formation despite the presence of gross differences in the hardness of materials.

While the location of palm trees has an effect on the differential accumulation of artefacts across the study area, the representation of raw materials closely follows the natural underlying local availability (figure 4, left). There is very little admixture of the two raw materials beyond what is reflected in the local distribution of stone. Tool material found at sites situated in areas with locally available fine-grained grey limestone comprises primarily fine-grained grey limestone. This pattern also holds for areas where red-oxidized limestone is locally abundant. However, in some assemblages, there are small proportions of red-oxidized limestone pieces represented; this is probably due to natural down-slope movement, given the asymmetry of the ravine.

By contrast, sites on the south-southeast side where red-oxidized limestone is predominately available comprise almost entirely this material. There are only three assemblages where fine-grained grey limestone is present within a red-oxidized limestone abundant area. One of these sites is situated within a few metres of the axis of the valley where there is the admixture of both limestones (figure 4, left). The other two are situated within a small drainage where isolated boulders of high-quality limestone are exposed.

The results of the binomial GLM show that the hardness of the available raw material had a significant effect on the proportion of high-quality grey limestone present in the assemblage. The full model was highly significant when compared with the null model (*X*^2^ = 11.526, d.f. = 29, *p*-value = 0.003). The estimated hardness of the available locally available was shown to have a significant positive effect on the proportion of grey limestone present within an assemblage (estimate: 0.18, *X^2^* = 12.28, *p*-value = 0.009). By contrast, the average distance to palm trees was also shown to have a positive effect on the proportion of high-quality grey limestone in an assemblage (estimate: 0.02). This result, however, was not statistically significant (*X^2^* = 7.55, *p*-value = 0.15).

### 3.4. Environmental influences on assemblage composition

Of the four assemblage attributes examined, only three showed significant correlations with the mean distance to palm trees or hardness. Only the number of fragments present within an assemblage showed a significant correlation with the average distance from palm trees (Kendall's tau, *τ* = −0.34, *p*-value = 0.005). The result showed that the number of fragments present within a site decreases as the average distance the assemblage is from a palm tree increases. This result further highlights that the incidental breakage of percussive implements plays an important role in the formation of nut-cracking assemblages. The representation of hammers and the number of flakes and flaked pieces, and the number of battered pieces is not significantly correlated with the average distance to a palm tree (electronic supplementary material, SOM 4).

Concerning the properties of the locally available material, the frequency of battered fragments is negatively correlated with hardness (Kendall's tau, *τ* = −0.33, *p*-value = 0.009). This implies that traces of battering are more frequent in assemblages where the material is softer (i.e. red-oxidized limestone). Moreover, the number of flakes and flaked pieces within an assemblage is significantly correlated with the hardness of locally available material (Kendall's tau, *τ* = 0.55, *p*-value < 0.0001). This implies that flakes and flaked pieces are predominantly present in assemblages that are in areas where high-quality grey limestone is locally available (figure 5). There are only three locations where flakes and flaked pieces were recovered from areas where red-oxidized limestone is locally available. Of these three locations, two are situated within a narrow drainage where high-quality limestone boulders are exposed. The third assemblage is situated close to the intersection of the ravine where both materials are available. Aside from these three assemblages, no flakes or flaked pieces are contained within samples collected from locations where red-oxidized material is immediately available. Thus, the presence of flakes and flaked pieces generally reflects the availability of the two general raw material types (figure 5).

## 4. Discussion

The integration of archaeological methods and theory with primate behaviour through the field of primate archaeology provides a powerful analytical framework for understanding how stone tool-using behaviours produce patterned material culture in a natural environment [42,79–81]. Our results show that the interaction of environmental variables with the nut-cracking behaviours of long-tailed macaques at ALP generates a compositionally diverse material landscape that varies in the density of artefacts, representation of raw materials, artefact types and attributes across space. The heterogeneity present in the stone assemblages is the result of an interaction between nut cracking, variation in the mechanical properties of stone material, and the distribution of food sources. Thus, the patterns described here allow us to discuss the linkage between tool-using behaviours, environmental circumstances, time and landscape-scale material patterning. The relationship between the pattern and process described at Lobi Bay, in turn, provides generalizable lessons about how artefact diversity may arise in the Plio-Pleistocene.

### 4.1. Macaque tool use, the environment and material culture

While macaques engage in short-distance bouts of transport to bring hammers to anvils, there is very little representation of materials in zones where they do not naturally occur. Aside from a single assemblage, there is no evidence of high-quality limestone outside of where it naturally occurs. As a result, the overall representation of raw materials within each nut-cracking assemblage reflects the availability of stone across the study area. Aside from a single assemblage, the consistency between tool material at nut-cracking sites and the local availability suggests that macaque tool transport does not have a considerable influence on the representation of tool stone in the material record. This might suggest that macaques exhibit little selectively or seldom transport specific materials beyond where they naturally occur. This pattern of transport is consistent with other studies of macaque tool use [82]

However, this pattern of transport does not indicate that macaques are incapable of selecting stones according to their physical properties. Previous studies of macaque tool selection indicate that they use stones of various morphologies depending on the task [48]. It is, therefore, more likely that this pattern is caused by the abundance of raw materials within the landscape. As suitable stones for use as hammers are abundantly available throughout the study area, there is little need to preferentially transport and use stones according to their material properties. This is probably further facilitated by the material properties of the nuts themselves. Palm nuts are relatively soft and thus can be cracked with any of the available stone materials in spite of the gross differences in their mechanical properties [83]. In this light, nut cracking at ALP may not require the selection of stones according to specific mechanical properties. Therefore, the results of this study potentially highlight the role of raw material abundance and the properties of the food resource in influencing the representation of rock types in the material record.

The lack of a significant correlation between the number of artefacts and the hardness of locally available stone indicates that raw material properties do not solely drive the accumulation of stone at nut-cracking sites. If the density of artefacts was driven by how breakable the rocks are, then one would expect the sites with the highest densities of material to be those where the breakable, red-oxidized material is most available. Instead, the greatest densities of material are found in places where the density of palm trees is highest, and the stone is least breakable. Palm trees ultimately dictate the availability of food resources at ALP. Therefore, a higher concentration of palm trees leads to a greater number of palm nuts available to consume. This would subsequently increase the frequency and potential duration of nut-cracking episodes, which lead to greater accumulations of material in areas where grey limestone is present. In this light, the significant correlation between artefact density and the average proximity to palm trees suggests that the continuous reuse of sites plays an important role in the accumulation of material at macaque nut-cracking sites. This result is further supported by the fact that the number of fragments present is significantly correlated with palm tree proximity, as it shows that the breakage of hammerstones through continuous use plays a major role in assemblage accumulation. Thus, the variation in the density of artefacts across ALP may reflect how often specific sites are used for nut cracking over time.

### 4.2. Implications for archaeology

The formational processes that give rise to the variability and structure in the macaque's material landscape at ALP also provide generalizable lessons about what can be inferred from archaeological variability. Archaeological interpretations of the past heavily rely on the ability to link variation in the technological and formal attributes of artefacts to aspects of hominin behaviour [9,14,15,36,48,84–87]. As research continues to push the origin of stone technology further back in time, it has become more critical to understand what the archaeological record may have looked like before the emergence of intentional core and flake technology [51,52]. The variability presented in this study, thus, highlights the role of material properties in influencing the range of forms that might be present in the earliest archaeological record.

Although the percussive elements associated with these assemblages often bear ubiquitous traces of damage [50,66], they are the least represented of all the technological categories. Given that these assemblages comprise largely fragments with some evidence of percussive damage, their archaeological visibility would be quite low as they would be hard to distinguish from naturally fragmented stones. Interestingly, however, some of the most archaeologically visible aspects of macaque nut cracking are those most likely confused with intentional flake production assemblages (e.g. hammerstones, flaked hammerstones and flakes) [50]. Therefore, these results provide useful insights into not only how patterning in percussive assemblages arises, but also help to calibrate our search lens for finding archaeological sites that extend beyond what is currently known.

The documented interactions between behaviour, the environment, and its material expression also have implications for behaviours inferred from Plio-Pleistocene stone tool variability. Stone tool variability is argued to reflect functional differences between sites [26], cognitive aptitude [11], social learning processes [14], raw material availability [71] and transport [31]. While archaeologists often use multiple lines of evidence from a variety of proxies to infer these various processes on archaeological patterning [88], the behavioural proxies for ESA behaviour are often restricted to patterning lithic assemblages [15]. In the absence of other proxies, the differences between the assemblages on either side of the ravine are so distinct that, if they were observed in an archaeological context, they may be considered to be two distinct behavioural processes, and that the material properties of the available stone influenced the functions for which the stone was used [89]. Yet, the material patterning at ALP emerges due to the repeated interaction of nut cracking with environmental factors dictating food resources and stone availability. Thus, the compositional variation in the material record at ALP, however, illustrates how the interaction of a single behaviour with the broader environmental factors can produce diverse material assemblages in the absence of functional, cognitive and/or social influences.

Within the broader field of social science, the term ‘emergence’ is used to describe structured patterns that are the result of a set of simple but interacting processes [90–92]. Research on the topic of emergence has begun to draw attention to how simple processes can create complex patterning [92,93]. Although many aspects of ecological and biological systems are considered emergent phenomena [92], the process of emergence is seldom discussed in Stone Age archaeology and often solely from a theoretical standpoint [94–97]. Since the past cannot be directly observed, understanding the role of emergence in shaping archaeological patterning is often limited to simulation or replicative experiments [15,35,36,95,98–100]. Thus, this work provides a real-world example of a material record whose patterning is an emergent property of an interaction between time, the environment, and behaviour. Therefore, this study highlights the potential role of emergence in producing observable patterning in the archaeological record. There is a general recognition that Plio-Pleistocene archaeological sites form due to a number of biotic and abiotic accumulating agents [16,101–103]. Therefore, the interaction of these various processes could produce a wider range of diversity than is currently considered.

Although the emergent patterning at ALP is specific to the percussive behaviour of macaques, the results of this study highlight the power of a few simple processes in producing material patterning. As a result, similar interactions could be responsible for diversity in the Plio-Pleistocene. For example, while some researchers have suggested that dense archaeological sites reflect functionally organized places such as home bases or campsites [23,24,27,104], others have suggested that they are merely preferred places that are continuously visited by hominins over time [101,105]. The results of this study provide support for the latter because they show how the density of materials can be influenced by proximity to food resources. This can, in turn, influence the frequency that a site is used over time. Therefore, it could be argued, aspects of hominin behavioural ecology such as habitat preference and access to food resources may play an important role in shaping the hominin record [17,106].

Moreover, the emergent nature of this record has broader implications for the spatio-temporal scale at which archaeological patterns become visible. The landscape material record at ALP does not reflect a set of functionally organized and temporally discrete behavioural episodes. Instead, the landscape patterning at ALP becomes visible through the aggregation of hundreds of individual episodes of nut cracking over time. Focusing on a single instance of nut cracking at ALP would show a small number of angular fragments, a hammerstone and possibly a few broken nut shells (if they are preserved). However, the patterns related to how the long-tailed macaques use this space would no longer be observable from the scale of a discrete behavioural episode. While Stone Age archaeologists often view the overprinting of behavioural events as a hindrance to reconstructing past behaviours [107], at ALP, it is the very process that allows us to draw connections between behaviour and measurable material patterning at the landscape scale. These results highlight the utility of investigating behavioural processes in the archaeological record at multiple spatial and temporal scales [22,63,89,108,109].

## 5. Conclusion

The processes that contribute to the variation in the Stone Age archaeological record are continuously debated. Primate archaeology provides a robust framework for linking behavioural ecology to the formation of the archaeological record. Our application of this framework to the nut-cracking macaques of Lobi Bay establishes a strong connection between the behavioural and environmental processes and the material patterning they produce. Thus, this work enhances our understanding of the behavioural ecological processes influencing nut cracking in long-tailed macaques. In doing so, our work shows that while macaque nut cracking presents a single percussive behaviour, its interaction with environmental factors such as raw material variability and resource distribution can produce a structured and diverse archaeological record. This work serves as support that the aggregate effects of a single behaviour can have profound effects on the diversity it produces. Therefore, our study not only highlights the relevance of primate archaeology for our understanding of macaque material culture but also for understanding the relationship between process and the formation of the archaeological record.

## Data Availability

The data and code necessary to reproduce the analysis presented in the paper are made available on the author's git-hub account. The data have been made accessible on the author's git-hub account at the following link. https://github.com/reevesj191/Macaque_Lithic_Landscapes_Reeves_et_al_Interface_2023. The data are provided in electronic supplementary material [110].
